# The Effects of Cadmium-Zinc Interactions on Biochemical Responses in Tobacco Seedlings and Adult Plants

**DOI:** 10.1371/journal.pone.0087582

**Published:** 2014-01-27

**Authors:** Mirta Tkalec, Petra Peharec Štefanić, Petra Cvjetko, Sandra Šikić, Mirjana Pavlica, Biljana Balen

**Affiliations:** 1 Department of Botany, Faculty of Science, University of Zagreb, Zagreb, Croatia; 2 Department of Molecular Biology, Faculty of Science, University of Zagreb, Zagreb, Croatia; 3 Department of Ecology, Institute of Public Health “Dr. Andrija Štampar”, Zagreb, Croatia; Dowling College, United States of America

## Abstract

The objective of the present study was to investigate the effects of cadmium-zinc (Cd-Zn) interactions on their uptake, oxidative damage of cell macromolecules (lipids, proteins, DNA) and activities of antioxidative enzymes in tobacco seedlings as well as roots and leaves of adult plants. Seedlings and plants were exposed to Cd (10 µM and 15 µM) and Zn (25 µM and 50 µM) as well as their combinations (10 µM or 15 µM Cd with either 25 µM or 50 µM Zn). Measurement of metal accumulation exhibited that Zn had mostly positive effect on Cd uptake in roots and seedlings, while Cd had antagonistic effect on Zn uptake in leaves and roots. According to examined oxidative stress parameters, in seedlings and roots individual Cd treatments induced oxidative damage, which was less prominent in combined treatments, indicating that the presence of Zn alleviates oxidative stress. However, DNA damage found in seedlings, and lower glutathione reductase (GR) and superoxide dismutase (SOD) activity recorded in both seedlings and roots, after individual Zn treatments, indicate that Zn accumulation could impose toxic effects. In leaves, oxidative stress was found after exposure to Cd either alone or in combination with Zn, thus implying that in this tissue Zn did not have alleviating effects. In conclusion, results obtained in different tobacco tissues suggest tissue-dependent Cd-Zn interactions, which resulted in activation of different mechanisms involved in the protection against metal stress.

## Introduction

Agricultural plants face a variety of abiotic and biotic stresses, which are major causes in limiting crop production. Among abiotic stressors, heavy metal contamination represents a global environmental problem endangering humans, animals and plants. All non-essential heavy metals, as well as essential ones, when present in higher concentrations than optimal, affect different cellular components, thereby interfering with the normal metabolic functions of plant cell. This can not only disturb redox homeostasis, but can also induce the accumulation of reactive oxygen species (ROS) [1]. Toxic levels of ROS can generate oxidative stress leading to inactivation and damage of proteins, membrane lipids and chlorophyll, and even DNA strand breaks [2]. To prevent the harmful effects of ROS, plants activate antioxidative enzyme systems, including superoxide dismutase (SOD), peroxidases like pyrogallol (PPX) and ascorbate peroxidase (APX) as well as catalase (CAT) and glutathione reductase (GR), and stimulate the production of antioxidant molecules such as ascorbic acid and glutathione [3].

Cadmium (Cd) is a very toxic divalent heavy metal cation and is non-essential for plants [4]. The toxic levels of Cd may be caused by natural soil characteristics or anthropogenic activities [5]. When released into the environment, Cd becomes accessible to plants, where it can induce complex changes at the genetical, biochemical and physiological levels, leading to phytotoxicity. The most prominent effects caused by Cd involve a growth inhibition and root damage [4], [6], reduction of water and nutrient uptake [7], lipid peroxidation [8], [9] and protein degradation [10], [11]. Toxicological properties of Cd could originate from its chemical similarity to zinc (Zn), an essential micronutrient in biological systems [12], although Cd could also interfere with an uptake, transport and use of several other elements [13]. Zn plays a fundamental role in numerous cellular functions [14], [15] and is involved in the catalytic function of many enzymes and structural stability of various cell proteins [16]. Zn also interacts in DNA/RNA binding by regulation of transcription, chromatin structure, protein-protein interactions and RNA metabolism [14]. Moreover, it has an important role in stabilization and protection of the biological membranes against oxidative and peroxidative injuries, loss of plasma membrane integrity and alteration of the membrane permeability [12]. Therefore, Zn deficiency can cause an increase in membrane permeability and a decrease in detoxification mechanisms [17], [18], which may be the major reason for impairment of various cellular functions in Zn-deficient plants. On the other hand, high levels of Zn inhibit many metabolic processes in plants, which can result in limited growth and root development and induce plant senescence [19], [20].

The biological effects of individual metals are more or less known, but even though combinations of heavy metals are common in nature, their combined effects still need to be thoroughly investigated [21], [22]. Cd frequently accompanies Zn minerals in the environment [23], and due to their chemical similarity they both can be taken by plants as divalent cations. It is well-known that metals in mixtures may act independently or interact to produce additive, synergistic, or antagonistic effects [21]. Studies conducted to investigate the Cd-Zn interaction on Cd and Zn uptake and accumulation have revealed mostly antagonistic interaction between these two metals [9], [12], [24], although synergistic effects were also reported [25], [26], [27]. Moreover, it was found that Zn supplementation in lower concentrations can decrease Cd-induced oxidative stress [9], [28], while high Zn level in combination with Cd can induce accumulation of oxidative stress, which suggests that higher Zn concentrations and Cd are synergistic in their effect on plant growth parameters and oxidative stress [15].

Plants may confront with different abiotic stresses during their lifespan, and different growth phases may have different responses to the stress [29]. The physiological strategies employed by the plant to cope with external toxic ions somewhat vary according to the specific developmental stage [30], [31]. It is, therefore, surprising that studies dealing with the physiological basis of heavy metal resistance most usually consider plant material at a single developmental stage. In our opinion comparative studies of young and adult plants may be important for abiotic stress tolerance research.

Therefore, the objective of the present study was to investigate the effects of Cd-Zn interactions on their uptake, oxidative damage of cell macromolecules (lipids, proteins, DNA) and activities of antioxidative enzymes (SOD, PPX, APX, CAT and GR) in tobacco seedlings as well as roots and leaves of adult plants.

## Materials and Methods

### Plant material and heavy metal treatments

Seeds of *Nicotiana tabacum* L. cv Burley were surface sterilized with 50% (v/v) NaOCl, washed with distilled H_2_O several times and subsequently germinated in sterilized nutrient medium. The medium was prepared according to Murashige and Skoog [32], with the addition of 500 mg L^−1^ MES [2-(N-morpholino)ethanesulfonic acid], 1.5 g L^−1^ sucrose and 2.2 g L^−1^ Phytagel (pH 5.6) [33], at 24°C with 16 h: 8 h light/dark cycle and light intensity of 90 µE m^−2^ s^−1^.

For heavy metal treatments Cd was applied in moderately high but environmentally relevant concentrations (10 and 15 µM), which have been investigated in our earlier studies [6], [9], [22], while selected Zn concentrations (25 and 50 µM) have previously been shown as effective in reducing Cd uptake and Cd-induced toxicity [9]. Tobacco seeds were germinated and grown for 30 days on the abovementioned solid MS medium with the addition of Cd, Zn or their combinations. Treatments with Cd were prepared by adding a stock solution of CdCl_2_ to the nutrient medium. Zn was added as ZnCl_2_ in the amounts suitable to achieve selected concentrations. Tobacco seedlings were also exposed to the combinations of metals (10 µM or 15 µM Cd with either 25 µM or 50 µM Zn). For analyses whole tobacco seedlings were used.

To obtain adult plants, tobacco seedlings were grown on the solid MS medium for three months under the abovementioned conditions until they fully developed root system and shoot with differentiated leaves. For exposure to heavy metals, plants were transferred to the liquid medium of the same composition but with the addition of either metal alone or their combinations, as it was applied for seedlings, and grown for 7 days. Leaves and roots were analyzed separately.

### Determination of Cd and Zn content

Samples were prepared following the standard EN 13657:2002 [34]. Plant material was oven-dried at 80°C for 24 h until a constant weight was reached. Subsequently, plant tissue was digested in microwave oven in two steps. The first step was digestion in 10 ml of 16 mM HNO_3_ at 70°C for 5 min, then at 130°C for another 5 min and finally at 150°C for 4 min. The second step included digestion in 1 ml of H_2_O_2_ at 85°C for 5 min and then at 130°C for 4 min. After cooling, the samples were diluted with 1% (v/v) HNO_3_ to achieve the total volume of 50 ml. For Cd and Zn analyses Inductively Coupled Plasma-Optical Emission Spectroscopy (ICP-OES, IRIS INTREPID II XSP) according to the standard EN ISO 11885:2009 [35] was used. The results were processed using TEVA software. Metal concentrations were calculated according to the calibration curve obtained with a set of standards of known concentrations. For Cd a lower concentration range of 1–50 µg kg^−1^ was used, while for Zn a higher concentration range of 50–5000 µg kg^−1^ was applied. Detection limits for Cd and Zn were 0.5 and 10 µg kg^−1^, respectively. Limit of quantification (LOQ) was <1 and <20 µg kg^−1^ for Cd and Zn, respectively.

### Malondialdehyde and carbonyl content

The level of lipid peroxidation was determined according to the modified method of Heath and Packer [36]. Fresh plant material (400 mg) was homogenized in 2 ml of 100 mM potassium phosphate buffer (pH 7.0), which contained 1 mM ethylenediaminetetraacetic acid (EDTA). After centrifugation at 20,000 g and 4°C for 20 min, the 200 µl of each supernatant was combined with 1300 µl of 0.3% (w/v) 2-thiobarbituric acid (TBA) in 10% (w/v) trichloroacetic acid (TCA). After heating at 95°C for 30 min, the mixture was cooled in an ice bath and centrifuged at 15,000 g for 10 min at 4°C. The absorbance of the supernatant was measured at 532 nm and correction for unspecific turbidity was done by subtracting the absorbance at 600 nm. As a blank 0.3% TBA in 10% TCA solution was used. The content of lipid peroxides was expressed as total 2-thiobarbituric acid reactive metabolites (TBARS), mainly malonyldialdehyde (MDA), as µmol g^−1^ of fresh weight using a molar absorption coefficient of 155 mM^−1^ cm^−1^.

For carbonyl quantification, the reaction with 2,4-dinitrophenylhydrazine (DNPH) was used as described by Levine et al. [37]. Fresh plant material (400 mg) was homogenized in 2 ml of 100 mM potassium phosphate buffer (pH 7.0). After centrifugation at 20,000 g and 4°C for 20 min, the 200 µl of each supernatant was combined with 300 µl of 10 mM DNPH in 2 M HCl. After 1 h incubation at room temperature, the proteins were precipitated with cold 10% (w/v) TCA and the pellets were washed three times with 500 µl of ethanol/ethylacetate (1/1 v/v) to remove excess reagent. The precipitated proteins were finally dissolved in 6 M urea in 20 mM potassium phosphate buffer (pH 2.4) and the absorption at 370 nm was measured. Protein recovery was estimated by measuring the absorbance at 280 nm. Carbonyl content was calculated using a molar absorption coefficient for aliphatic hydrazones of 22 mM^−1^ cm^−1^ and expressed as µmol per mg of protein.

### Comet assay

The procedure was carried out according to Gichner et al. [38]. In brief, nuclei were mechanically isolated in 400 mM Tris-HCl (pH 7.5) at 4°C, mixed in equal amounts (50 µL) with low melting point agarose (LMP, 1% (w/v) in PBS) preheated at 42°C, spread onto partially frosted microscope slides precoated with 1% (w/v) NMP (normal melting point) agarose in distilled water, covered with cover slip and left on ice for solidification of agarose. Prior to electrophoresis, the cover slip was removed and the slides were placed in horizontal electrophoresis tank filled with freshly prepared chilled electrophoresis buffer (1 mM Na_2_EDTA and 300 mM NaOH, pH>13) for 10 min for DNA unwinding. Following denaturation, electrophoresis was done in the same buffer for 20 min at 0.8 V cm^−1^ and 300 mA. After electrophoresis slides were gently rinsed three times for 5 min with neutralization buffer (400 mM Tris-HCl buffer, pH 7.5) and finally briefly dipped in distilled water.

For analysis the slides were stained with 70 µL ethidium bromide (20 µg mL^−1^) for 5 min. For each slide, 150 randomly chosen nuclei per each treatment were analyzed under a fluorescence microscope (Zeiss Axioplane) equipped with an excitation filter BP 520/09 nm and a barrier filter of 610 nm. A computerized image analysis system (Komet version 5, Kinetic Imaging Ltd., Liverpool, UK) was employed to measure the percentage of tail DNA (% tDNA) as the primary measure of DNA damage.

In the comet test, fragmented DNA forms the structure resembling the comet tail and therefore percentage of tail DNA was used as a measure of DNA damage.

### Assays of enzymatic activities

Total soluble proteins were extracted by grinding 40 mg of fresh plant tissue in 1.5 ml of 100 mM potassium phosphate buffer, pH 7.0. The buffer used for extraction of APX was supplemented with 0.5 mM ascorbate. The insoluble polyvinylpyrrolidone (10 mg) was added to tissue samples prior to extraction. The homogenates were centrifuged at 20,000 g and 4°C for 15 min. Obtained supernatants were centrifuged again for 60 min at 20,000 g at 4°C. Supernatants were collected and protein content was determined according to Bradford [39] using bovine serum albumin as a standard and were subsequently used for the following enzyme assays.

SOD (E.C. 1.15.1.1) activity was determined by measuring inhibition of the photochemical reduction of nitroblue tetrazolium (NBT) using the method of Beauchamp and Fridovich [40]. The reaction mixture was composed of 13 mM methionine, 75 mM NBT, 0.1 mM EDTA, 0.002 mM riboflavin, and different volumes of enzyme extract in 50 mM phosphate buffer (pH 7.8). The mixture was placed in a light box (15 W) for 8 min. The increase in absorbance due to formazan formation was read at 560 nm. One unit of SOD activity was defined as the amount of enzyme required to cause 50% inhibition of the rate of NBT reduction at 560 nm. Activity was expressed as units of SOD activity per mg of protein.

PPX (E.C. 1.11.1.7) activity was assayed by monitoring the increase in absorbance at 430 nm due to the oxidation of pyrogallol (ε = 2.6 mM^−1^ cm^−1^), as described by Nakano and Asada [41]. The reaction mixture consisted of 50 mM potassium phosphate buffer (pH 7.0), 20 mM pyrogallol, 1 mM H_2_O_2_, and 20 µl enzyme extract. PPX activity was expressed as µmol of purpurogallin (product of pyrogallol oxidation) per min per mg of protein.

APX (E.C. 1.11.1.11) activity was determined by the decrease in absorbance at 290 nm (ε = 2.8 mM^−1^ cm^−1^), as described by Nakano and Asada [41]. The reaction mixture consisted of 50 mM potassium phosphate buffer (pH 7.0), 0.1 mM ascorbate, 0.12 mM H_2_O_2_, and 180 µl enzyme extract. APX activity was expressed as µmol of oxidized ascorbate per min per mg of protein.

CAT (E.C. 1.11.1.6) activity was assayed by measuring the decrease in absorbance at 240 nm (ε = 36 mM^−1^ cm^−1^), according to Aebi [42]. The reaction mixture consisted of 50 mM potassium phosphate buffer (pH 7.0), 10 mM H_2_O_2_, and 50 µl enzyme extract. CAT activity was expressed as µmol of decomposed H_2_O_2_ per min per mg of protein.

GR (E.C. 1.8.1.7) was assayed according to the method of Foyer and Halliwell [43] by following the decrease in absorbance at 340 nm due to NADPH oxidation (ε = 6.2 mM^−1^ cm^−1^). The reaction mixture consisted of 50 mM potassium phosphate buffer (pH 7.0), 0.1 mM EDTA, 0.5 mM oxidized glutathione (GSSG), 0.12 mM nicotinamide adenine dinucleotide phosphate (NADPH), and the enzyme aliquot. Corrections were made for NADPH oxidation in the absence of GSSG. GR activity was expressed as µmol of oxidized NADPH per min per mg of protein.

### Statistical analysis

The results of each assay were compared by the analysis of variance (ANOVA) and the least significant difference (LSD) test was performed if ANOVA was significant at P<0.05. The analysis was done for seedlings, roots and leaves data sets separately. Each data point is the average of six replicates.

Principal component analysis (PCA) was performed to evaluate the most important responses in different tobacco tissues exposed to Cd and Zn as well as to discriminate the responses to individual metals and mixtures. The seedlings (S), roots (R) and leaves (L) data sets used for PCA were comprised of 10 (Cd accumulation, Zn accumulation, MDA and protein carbonyl content, activity of SOD, APX, PPX, CAT and GR as well as DNA damage) variables. PCA was applied to the standardized data sets and the factor loadings were classified as ‘strong’, ‘moderate’, and ‘weak’ corresponding to absolute loading values of >0.75, 0.75–0.50, and 0.50–0.30, respectively [44].

Statistical analyses were done using the STATISTICA 8.0 (Stat Soft Inc., USA) software package.

## Results

### Cd and Zn content

In all investigated tobacco tissues, which were not exposed to Cd (control and Zn treated), detected Cd content was below the instrument LOQ (<0.001 µg g^−1^). The lowest Cd content measured in seedlings was obtained after treatment with 10 µM Cd alone, while the highest values were obtained in seedlings exposed to individual treatment with 15 µM Cd (Table 1). In combined treatments of 10 µM Cd with either 25 or 50 µM Zn, the Cd content doubled in comparison to individual treatment with 10 µM Cd. On the contrary, in combinations of 15 µM Cd with both Zn concentrations, Cd content was slightly reduced in comparison to 15 µM Cd alone, although it was not a statistically significant difference. Measurements of Zn content revealed that individual Cd treatments did not exhibit significant effect on Zn uptake when compared to control. Significantly the highest Zn uptake was recorded in combined treatments of 10 µM Cd with either 25 or 50 µM Zn, while combinations of 15 µM Cd with both Zn concentrations exhibited similar values as individual treatments with 25 or 50 µM Zn (Table 1).

**Table 1:** Cd and Zn content and their effects on MDA and protein carbonyl contents and DNA in tobacco seedlings after exposure to Cd (10 and 15 µM), Zn (25 and 50 µM) and their combinations.

Treatment	Cd content µg g^−1^_DW_	Zn content µg g^−1^_DW_	MDA content µmol g^−1^_FW_	Carbonyl content µmol mg^−1^_protein_	% tail DNA
**control**	<0.001^d^	171.4±9.3^c^	186.29±30.23^bc^	50.12±3.87^c^	2.95±0.73^e^
**10 µM Cd**	102.5±12.1^c^	125.8±8.9^c^	237.23±20.59^ab^	69.73±6.20^ab^	7.45±1.29^bc^
**15 µM Cd**	261.5±17.8^a^	100.9±7.2^c^	259.74±40.78^a^	71.94±2.87^a^	7.13±1.67^cd^
**25 µM Zn**	<0.001^d^	240.4±5.0^bc^	184.36±13.63^bc^	53.30±4.13^c^	7.49±0.63^bc^
**50 µM Zn**	<0.001^d^	311.7±6.5^ab^	203.99±24.05^abc^	54.76±7.80^c^	7.93±0.66^abc^
**10 µM Cd 25 µM Zn**	204.0±6.1^ab^	356.4±3.8^a^	169.66±9.78^c^	55.43±6.15^c^	10.63±1.36^a^
**10 µM Cd 50 µM Zn**	209.0±6.5^ab^	354.5±3.0^a^	219.01±12.67^abc^	55.52±5.07^c^	10.04±0.54^ab^
**15 µM Cd 25 µM Zn**	212.5±3.2^ab^	261.1±21.0^bc^	193.71±9.37^bc^	59.94±3.58^abc^	9.83±0.65^abc^
**15 µM Cd 50 µM Zn**	235.7±3.9^ab^	279.8±4.6^b^	193.03±12.23^bc^	62.70±3.63^abc^	4.54±0.56^de^

Values represent means ± standard errors (n = 6). Values marked with different letters indicate significantly different treatments (P<0.05) within seedling data set (LSD multiple test).

In roots, similar accumulation of Cd was measured in both individual Cd treatments. Moreover, higher Cd uptake was revealed in combined treatments in comparison to individual ones; significantly the highest Cd content was measured in combination of 50 µM Zn with either 10 or 15 µM Cd, while the treatment of 15 µM Cd with 25 µM Zn resulted with higher Cd uptake in comparison to individual Cd treatments. The Zn content was significantly reduced after both individual Cd treatments in comparison to control. The highest Zn uptake was recorded after exposure to 50 µM Zn alone or in combination with 10 µM Cd, while in the combined treatment of 15 µM Cd and 50 µM Zn it was significantly lower. Individual treatment with either 25 or 50 µM Zn gave similar results as their combination with 10 µM Cd; however, in combination with 15 µM Cd the Zn uptake was significantly reduced (Table 2). In leaves, significantly higher Cd content was obtained after treatment with 15 µM Cd in comparison to treatment with 10 µM Cd. In all combined treatments Cd content was reduced in comparison to respected individual treatments, although obtained values were not statistically significant except for the combined treatment with 15 µM Cd and 50 µM Zn. Zn uptake in leaves was not affected in individual Cd treatments. The highest Zn content was measured after individual treatment with 50 µM Zn, while its combinations with either 10 or 15 µM Cd resulted with significantly reduced values.

**Table 2:** Cd and Zn content and their effects on MDA and protein carbonyl contents and DNA in roots and leaves of adult tobacco plants after exposure to Cd (10 and 15 µM), Zn (25 and 50 µM) and their combinations.

Treatment	Root					Leaf				
	Cd content µg g^−1^_DW_	Zn content µg g^−1^_DW_	MDA content µmol g^−1^_FW_	Carbonyl content µmol mg^−1^_protein_	% tail DNA	Cd content µg g^−1^_DW_	Zn content µg g^−1^_DW_	MDA content µmol g^−1^_FW_	Carbonyl Content µmol mg^−1^_protein_	% tail DNA
**control**	<0.001^D^	1772.0±62.5^D^	169.51±7.76^D^	55.04±5.78^C^	4.59±1.45^B^	<0.001^d^	151.0±4.4^d^	277.11±21.44^d^	25.12±0.92^c^	3.76±0.54^c^
**10 µM Cd**	723.4±12.5^C^	980.7±57.1^E^	273.65±30.05^BC^	148.45±14.61^A^	9.34±1.70^A^	24.4±1.6^bc^	163.1±19.5^d^	439.07±27.99^ab^	33.05±2.14^bc^	8.79±1.08^a^
**15 µM Cd**	738.4±13.5^BC^	815.1±39.7^E^	390.24±28.7^ A^	148.25±24.31^A^	9.97±2.28^A^	35.3±2.6^a^	160.9±17.6^d^	448.24±32.54^ab^	33.28±4.28^bc^	9.73±1.84^a^
**25 µM Zn**	<0.001^D^	2591.0±88.2^B^	217.84±23.99^CD^	85.79±16.55^BC^	5.25±2.03^B^	<0.001^d^	381.1±40.2^ab^	319.94±38.02^cd^	25.22±1.42^c^	4.53±0.39^bc^
**50 µM Zn**	< 0.001^D^	2984.0±94.1^A^	274.33±19.07^BC^	109.81±17.82^AB^	7.43±1.33^AB^	< 0.001^d^	423.4±44.9^a^	379.74±30.64^bc^	25.26±2.30^c^	6.15±0.46^abc^
**10 µM Cd 25 µM Zn**	727.0±26.5^C^	2674.0±75.3^B^	271.57±24.40^BC^	82.06±14.74^BC^	5.10±0.64^B^	24.2±2.1^bc^	346.7±3.7^b^	494.00±32.96^a^	36.98±2.53^b^	7.91±1.51^ab^
**10 µM Cd 50 µM Zn**	826.1±29.2^A^	2978.0±48.6^A^	233.75±16.01^C^	83.31±7.94^BC^	4.76±0.63^B^	20.8±1.1^c^	341.2±6.5^b^	485.75±36.51^a^	36.50±4.47^b^	8.63±1.74^a^
**15 µM Cd 25 µM Zn**	757.1±22.3^B^	1747.0±50.3^D^	248.09±19.36^C^	144.98±10.47^A^	5.15±1.06^B^	32.5±0.9^ab^	333.9±31.9^bc^	478.61±18.20^a^	48.38±5.78^a^	8.80±1.96^a^
**15 µM Cd 50 µM Zn**	839.1±18.4^A^	2304.8±36.2^C^	325.14±20.29^B^	97.80±9.75^B^	5.97±0.32^AB^	31.3±6.1^b^	343.5±9.6^b^	418.52±58.64^ab^	39.06±6.59^ab^	7.90±1.57^ab^

Values represent means ± standard errors (n = 6). Values marked with different capital letters indicate significantly different treatments (P < 0.05) within root data set, while values marked with different lowercase letters indicate significantly different treatments (P < 0.05) within leaf data set (LSD multiple test).

### Effect on lipid peroxidation and protein oxidation

In seedlings, treatment with 15 µM Cd resulted with the highest MDA content, although combined treatment with either 25 or 50 µM Zn significantly reduced the values. Moreover, addition of 25 µM Zn to 10 µM Cd significantly decreased lipid peroxidation when compared to values obtained after exposure to 10 µM Cd alone (Table 1). The highest carbonyl content was measured after exposure to both individual Cd treatments, while other treatments revealed values that were not significantly different in comparison to control (Table 1). However, after exposure to combined treatments of 10 µM Cd with either 25 or 50 µM Zn, significantly reduced protein oxidation was recorded in comparison to individual Cd treatment.

Roots of plants exposed to 15 µM Cd exhibited the highest MDA content, which was significantly reduced in combined treatments with 25 and 50 µM Zn (Table 2). Enhanced lipid peroxidation was also recorded in all other treatments in comparison to control, except in the one with 25 µM Zn. In leaves, enhanced lipid peroxidation was recorded in all treatments compared to control, except the one with 25 µM Zn, which resulted with values not significantly different from control. The highest MDA values were recorded in all combined treatments (Table 2).

As for the carbonyl content, treatments with both individual Cd concentrations resulted in the significantly highest values in roots compared to control, which was also recorded after exposure to 50 µM Zn as well as to combination of 15 µM Cd and 25 µM Zn. Significantly higher protein oxidation was also observed upon combined treatment with 15 µM Cd and 50 µM Zn (Table 2), but values were significantly lower compared to individual treatment with 15 µM Cd. Both Zn concentrations alleviated the toxic effect of 10 µM Cd in combined treatments, while only 50 µM Zn significantly decreased carbonyl content in combination with 15 µM Cd. Leaves responded with significantly higher values only after growth on all combined treatments compared to control (Table 2). Moreover, higher protein oxidation was obtained after exposure to combination of 15 µM Cd and 25 µM Cd than in respective individual Cd treatment.

### Effect on DNA

All individual treatments with either Cd or Zn exhibited significant increase in DNA damage as percentage in tail DNA (% tail DNA) when compared to control seedlings (Table 1). However, in almost all combined treatments significantly higher DNA damage was recorded in comparison to exposure to respective individual Cd or Zn concentrations.

Roots exhibited more DNA damage only after individual treatments with both Cd concentrations, while in leaves the increase in % tail DNA was observed after both individual Cd as well as after all combined treatments (Table 2).

### Effect on antioxidant enzyme activity

**Figure 1:**
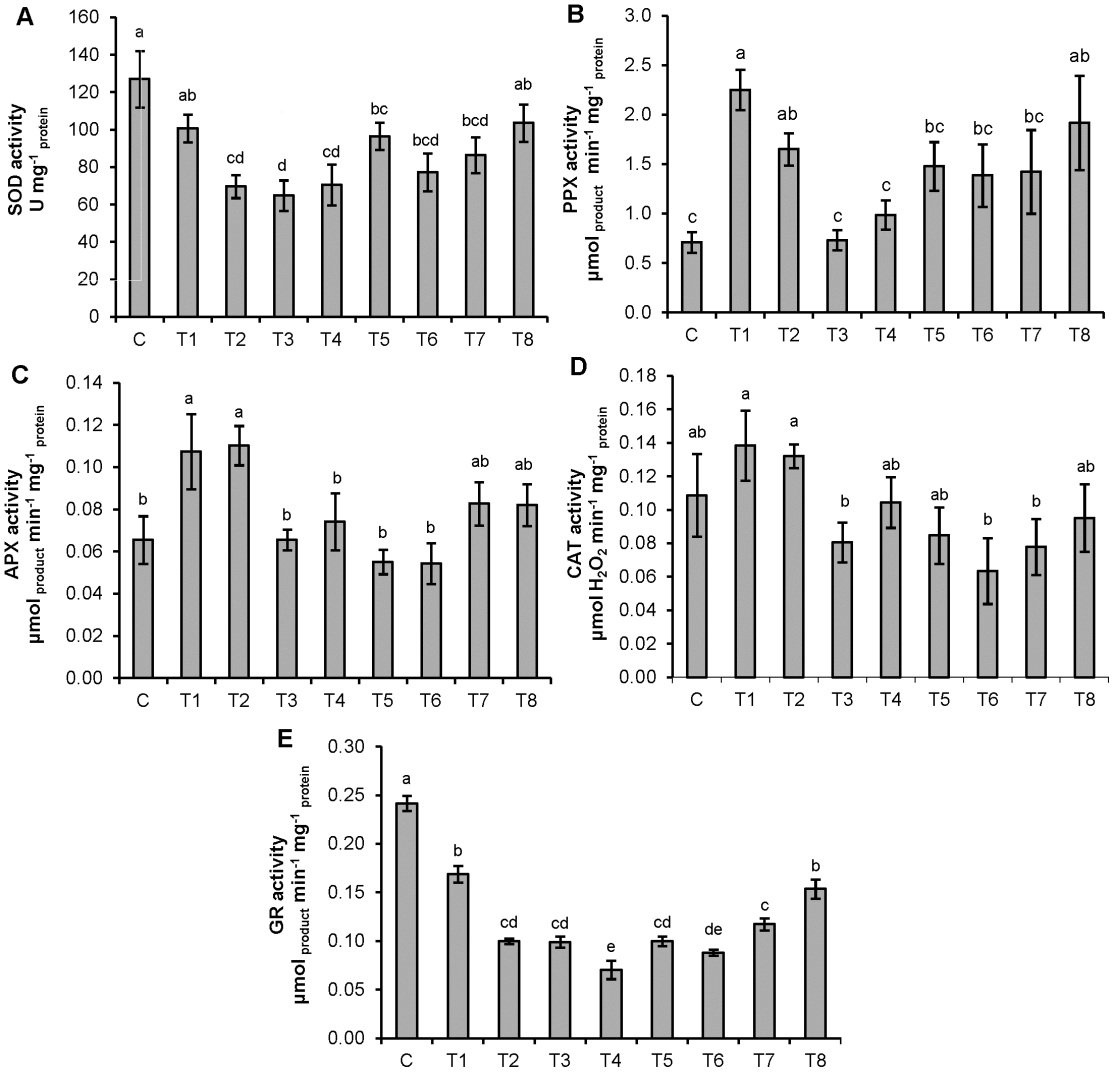
Activities of antioxidative enzymes in tobacco seedlings. (A) SOD, (B) PPX, (C) APX, (D) CAT and (E) GR. Treatments: C – control, T1 – 10 µM Cd, T2 – 15 µM Cd, T3 – 25 µM Zn, T4 – 50 µM Zn, T5 – 10 µM Cd and 25 µM Zn, T6 –10 µM Cd and 50 µM Zn, T7 – 15 µM Cd and 25 µM Zn, T8 – 15 µM Cd and 50 µM Zn. Vertical bars represent standard errors (n = 6). Columns marked with different letters indicate significantly different treatments (P<0.05) within seedling data set (LSD multiple test).

The highest SOD activity was measured in control seedlings, while individual as well as combined treatments resulted with lower values (Figure 1A). The highest PPX activity was recorded in seedlings grown on a medium containing 10 µM Cd, while addition of either 25 or 50 µM Zn significantly reduced obtained values. Individual treatment with 15 µM Cd as well as its combination with 50 µM Zn resulted with values significantly higher than control (Figure 1B). Enhanced APX activity was recorded on both individual Cd treatments, while addition of either Zn concentration significantly lowered obtained values only in combination with 10 µM Cd when compared to respective Cd treatment. Exposure to individual Zn concentrations resulted with values which were not significantly different from the control (Figure 1C). No significant difference was found in CAT activity between control and treated tobacco seedlings (Figure 1D), while all applied treatments significantly decreased GR activity (Figure 1E).

**Figure 2:**
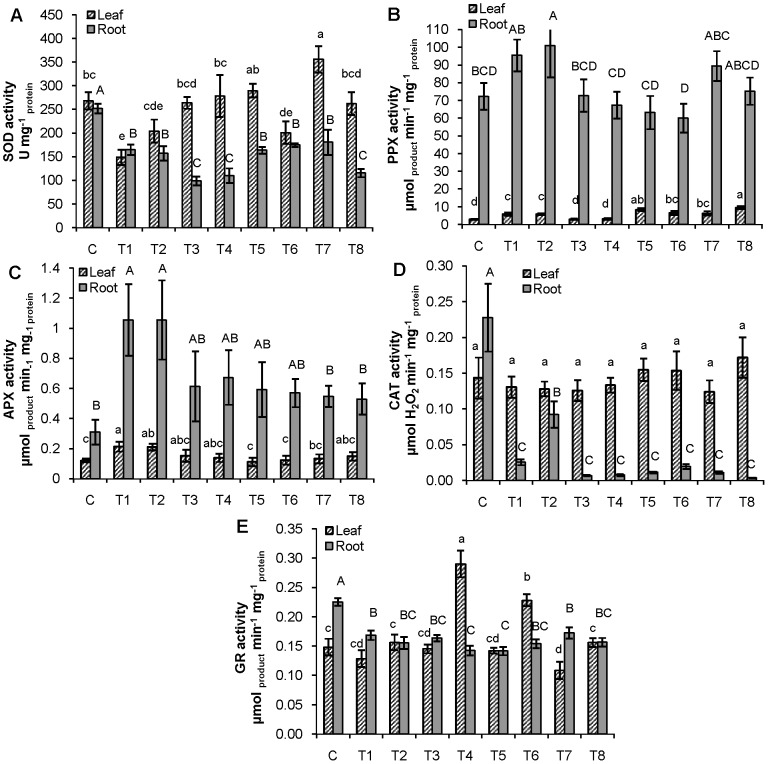
Activities of antioxidative enzymes in leaves and roots of adult tobacco plants. (A) SOD, (B) PPX, (C) APX, (D) CAT and (E) GR. For explanation of treatments see Fig. 1. Vertical bars represent standard errors (n = 6). Columns marked with different lowercase letters indicate significantly different treatments (P<0.05) within leaf data set, while columns marked with different capital letters indicate significantly different treatments (P<0.05) within root data set (LSD multiple test).

In roots of adult plants, all applied treatments exhibited significantly lower SOD activity when compared to control (Figure 2A), which was particularly pronounced in individual Zn treatments as well as in combination of 15 µM Cd and 50 µM Zn. In leaves, both individual Cd treatments as well as the combination of 10 µM Cd with 50 µM Zn significantly decreased the SOD activity compared to control (Figure 2A), while those treated with combinations of 10 or 15 µM Cd with 25 µM Zn showed higher SOD activity in comparison to individual Cd treatments.

Roots exhibited significantly higher PPX activity only after exposure to 15 µM Cd when compared to control (Figure 2B). After treatments with combination of both Zn concentrations and 10 µM Cd activity was lower compared to individual treatment with 10 µM Cd (Figure 2B). In leaves, enhanced PPX activity was measured upon exposure to both individual Cd concentrations in comparison to control, although all combined treatments induced even higher PPX activity (Figure 2B).

In both roots and leaves, significantly enhanced APX activity was recorded only upon treatments with either 10 or 15 µM Cd (Figure 2C). In roots, both Zn concentrations lowered activities when applied with 15 µM Cd, while in leaves activity decreased after treatment with combinations of both Zn concentrations and 10 µM Cd (Figure 2C).

All treatments decreased CAT activity in roots compared to control, while no significant difference in CAT activity was found between control and leaves of treated plants (Figure 2D).

All applied treatments significantly decreased GR activity in roots, while in leaves exposed to 50 µM Zn, either alone or in combination with 10 µM Cd, significantly the highest GR activity was measured (Figure 2E).

### PCA analysis

**Figure 3:**
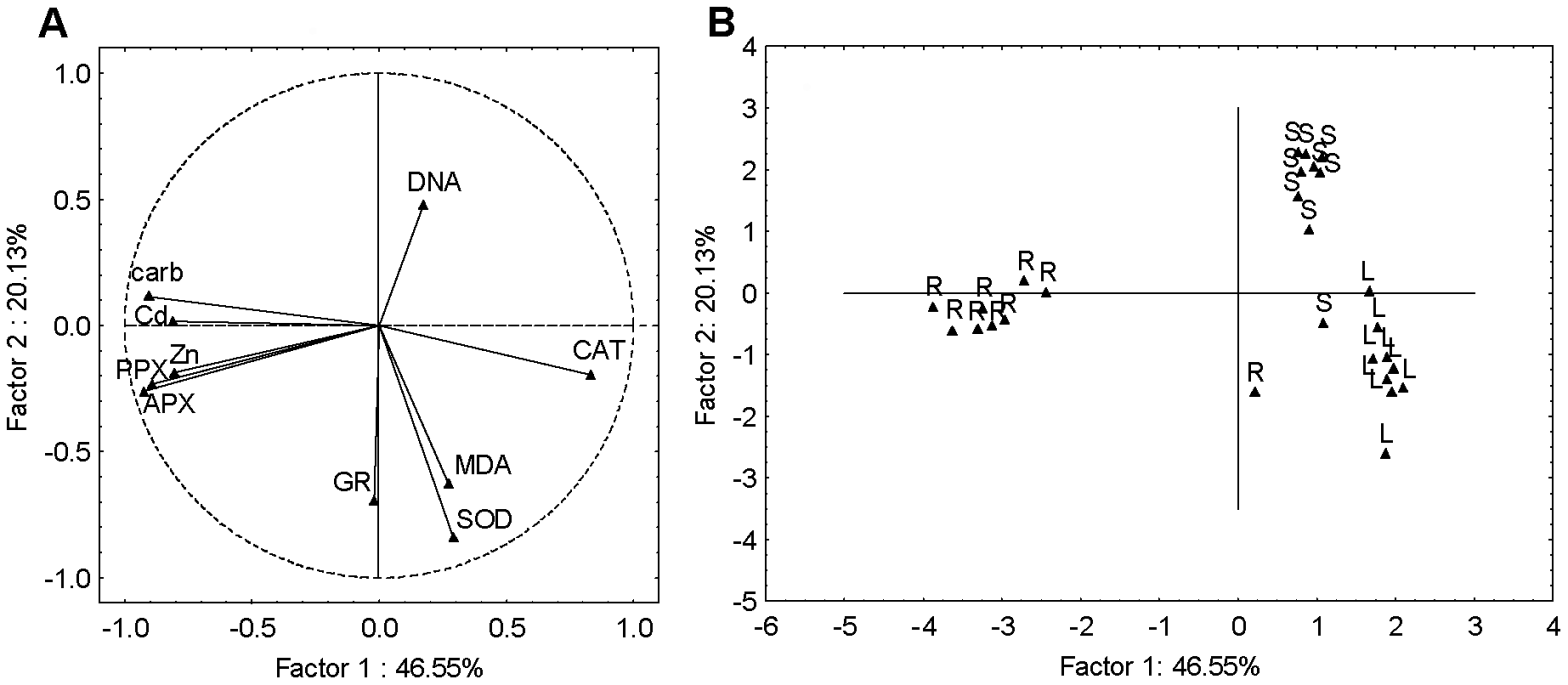
Principal component analysis of combined seedlings (S), roots (R) and leaves (L) data sets. (A) loadings and (B) scores of the first two factors (PCs). Cd accumulation (Cd), Zn accumulation (Zn), MDA content (MDA), carbonyl content (carb), DNA damage (DNA) and activity of SOD, APX, PPX, GR and CAT represent variables.

Firstly, PCA was applied to the combined seedlings, roots and leaves data sets yielding three significant PCs capturing about 80% of the total data variance. The first two PCA components (PC1 and PC2) explained 67% of the total variance (Figure 3A). The PC1 (47%) was largely determined by Cd and Zn accumulation, carbonyls, PPX and APX activity with strong negative loadings and CAT activity with strong positive loading. The PC2 (20%), had moderate positive loading on DNA damage and strong negative loading on SOD activity as well as moderate negative loadings on GR activity and MDA content. The scores plot (Figure 3B) suggested differentiation between the seedlings, roots and leaves responses, grouping them into three visible clusters. It may be noted that the roots responded in terms of carbonyls, PPX and APX activity as well as Zn and Cd accumulation, seedlings in term of DNA, whereas response in leaves was more prominent in terms of SOD and GR activity as well as MDA content.

Furthermore, the differences between the responses of seedlings, roots and leaves of the plants exposed to different metal treatments were investigated through PCA applied to the normalized data sets of the seedlings, roots and leaves, separately.

**Figure 4:**
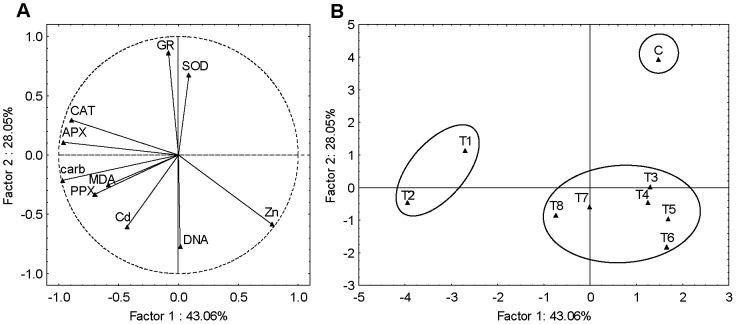
Principal component analysis of seedlings data set. (A) loadings and (B) scores of the first two factors (PCs). Cd accumulation (Cd), Zn accumulation (Zn), MDA content (MDA), carbonyl content (carb), DNA damage (DNA) and activity of SOD, APX, PPX, GR and CAT represent variables. For explanation of treatments see Fig. 1.

In seedlings, the three significant PCs represented 91% of the total variance. The PC1 (43%) correlated strongly with Zn accumulation in the positive part and with the carbonyls, APX and CAT activity in the negative part. The PC2 (28%) was strongly correlated with GR activity and moderately correlated with SOD in positive part, while strong negative correlation was found with DNA damage and moderate negative correlation with Cd and Zn accumulation (Figure 4A). From the scores plot (Figure 4B) it can be noted that exposures to Cd-alone (T1 and T2) differ from all other treatments as well as control, exhibiting changes in carbonyls, APX and CAT activity and Zn content. Furthermore, combined treatments, T5 and T6 (mixtures with 10 µM Cd), T7 and T8 (mixtures with 15 µM Cd) as well as T3 and T4 (individual Zn treatments) grouped together in a cluster which separated from control, being characterized with DNA damage as well as Zn and/or Cd accumulation.

**Figure 5:**
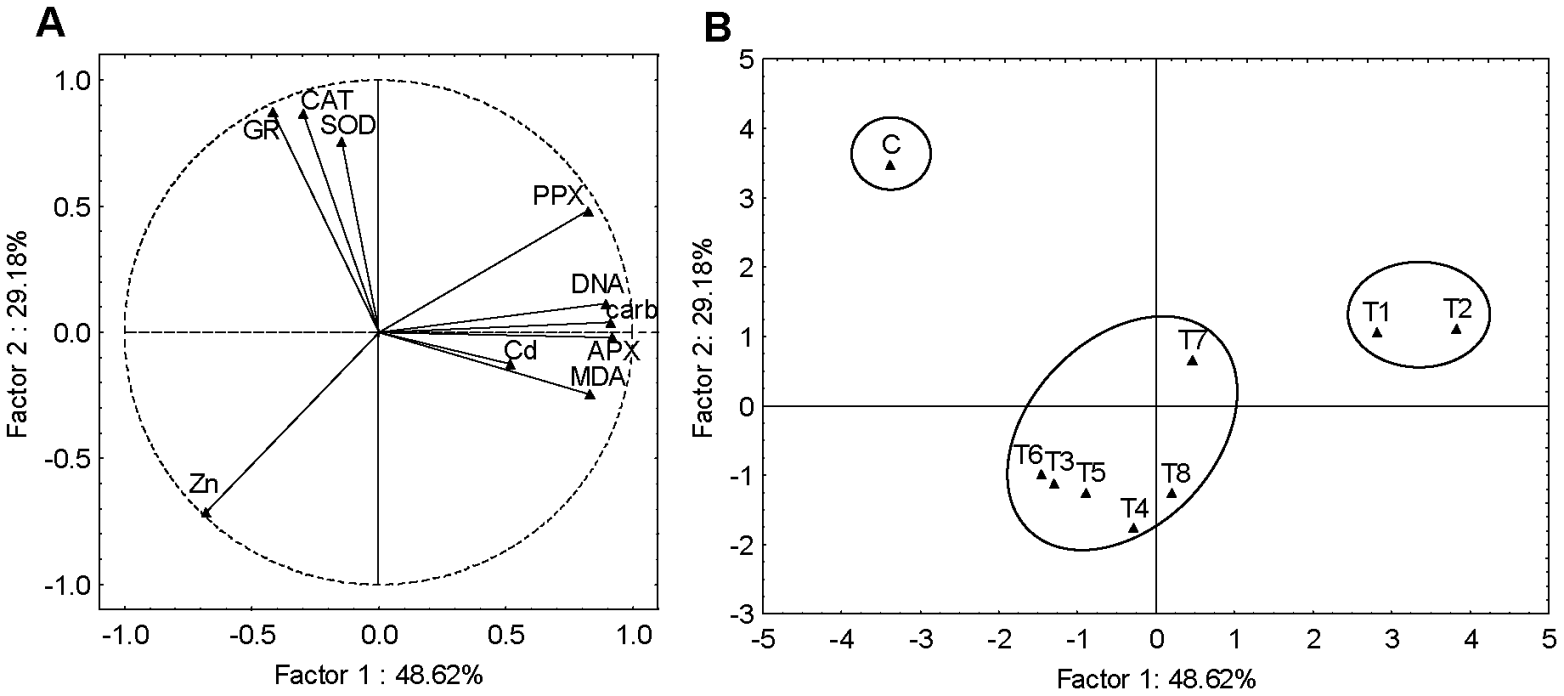
Principal component analysis of roots data set. (A) loadings and (B) scores of the first two factors (PCs). Cd accumulation (Cd), Zn accumulation (Zn), MDA content (MDA), carbonyl content (carb), DNA damage (DNA) and activity of SOD, APX, PPX, GR and CAT represent variables. For explanation of treatments see Fig. 1.

PCA performed on root data set yielded three significant PCs explaining 90% of data variance. It may be noted that PC1 (49%) showed high positive loadings on carbonyls, MDA content, DNA damage, PPX and APX activity as well as moderate positive loadings on Cd accumulation and moderate negative loadings on Zn accumulation. PC2 (29%) showed strong positive loadings on CAT, GR and SOD activity as well as a moderate negative loadings on Zn accumulation (Figure 5A). The corresponding scores plot (Figure 5B) in conjunction with the loadings plot (Figure 5A) suggest that roots exposed to individual Cd treatment differ from all other treatments, particularly control, exhibiting influence on carbonyls, MDA content, DNA damage, PPX and APX activity and correlating negatively with Zn accumulation. Exposures to individual Zn treatments (T3, T4) and combined treatments (T5, T6, T7 and T8) differ from control plants exhibiting significant changes in CAT, GR and SOD activity and moderate Zn accumulation.

**Figure 6:**
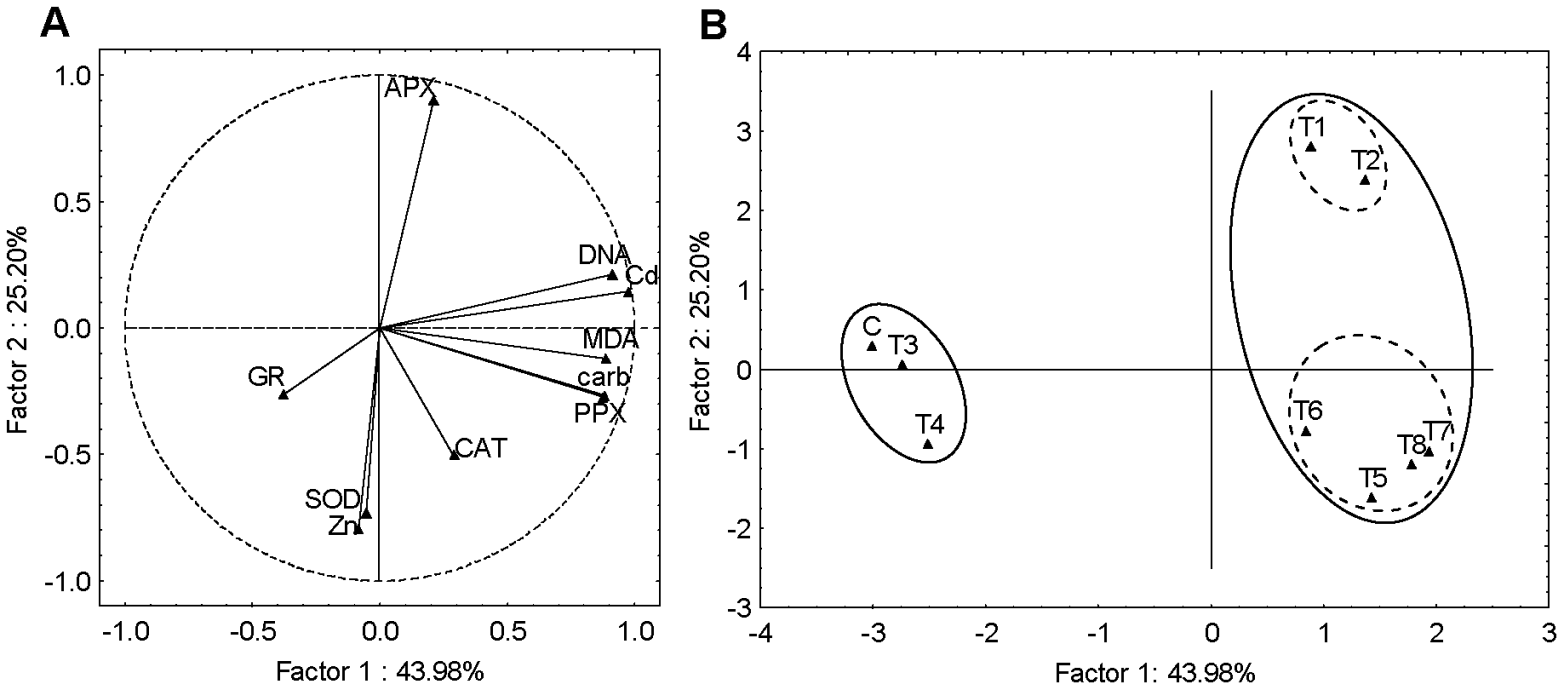
Principal component analysis of leaves data set. (A) loadings and (B) scores of the first two factors (PCs). Cd accumulation (Cd), Zn accumulation (Zn), MDA content (MDA), carbonyl content (carb), DNA damage (DNA) and activity of SOD, APX, PPX, GR and CAT represent variables. For explanation of treatments see Fig. 1.

PCA of the leaves data set yielded four significant PCs explaining 94% of the total variance. It is evident that PC1 (44%) is largely determined by Cd accumulation, carbonyls, MDA content, DNA damage and PPX activity having strong positive loadings. On the other hand, PC2 (25%) has a strong negative loadings on Zn accumulation and SOD and strong positive loadings on APX (Figure 6A). From the scores plot (Figure 6B) it can be noted that treatments without Cd (control – C and individual Zn treatments – T3 and T4) differ from treatments with Cd, applied either alone (T1 and T2) or as the mixtures (T5, T6, T7 and T8), later being characterized with Cd accumulation, carbonyls, MDA content, DNA damage and PPX activity. Moreover, this cluster can be further separated in two distinct groups, one representing Cd-alone treatments (T1 and T2) and one representing treatments with Cd/Zn mixture (T5, T6, T7 and T8). These groups differentiated in Zn accumulation and SOD activity as well as in APX activity.

## Discussion

The present investigation has shown that Cd and Zn both tend to accumulate in tobacco seedlings and plants, which is in accordance with the data reported for *Nicotiana* [38], [45] as well as for other plant species [15], [46]. The higher Cd and Zn concentration in the growth medium resulted in an enhanced metals uptake [47], [48], although the metal content obtained in tobacco roots was several times higher than in leaves. Namely, despite the different mobility of metal ions in plants, the metal content is generally higher in roots than in the above-ground tissues [49]. In most environmental conditions, Cd accumulates in the roots because plants reduce its translocation to the shoots in order to prevent metal induced damage in the shoots. Although majority of studies found antagonistic effects of these two metals, an analysis of Cd-Zn interaction mechanism in some plant species indicated that the effects could be synergistic as well [25], [26], [27]. Namely, the laboratory investigations are usually performed under fully controlled conditions and obtained results can only partly be used to predict the effects under environmental conditions, which are much more complex and unstable. In this study, among combined treatments, positive or no influence of Cd and Zn on each other uptake was recorded in tobacco seedlings, which suggests synergistic effect of Cd and Zn. In roots exposed to combined treatments of 10 or 15 µM Cd with 50 µM Zn, higher Cd uptake was observed in comparison to respective individual treatments, which indicated that Zn stimulated Cd uptake. Significant increases in root Cd concentrations due to Zn addition were also noticed in *Sedum alfredii* Hance [50]. In tobacco leaves, Zn had no significant influence on Cd uptake in combined treatments, while Cd had a significant effect on reduction of Zn content when compared to respected individual treatments. The similar antagonistic impact of Cd on Zn uptake was also observed in roots treated with 15 µM Cd with either 25 or 50 µM Zn. Moreover, roots exhibited reduced Zn uptake in both individual Cd treatments. Previously, it was reported that Cd addition significantly decreased Zn concentrations in plant tissues, especially in roots [51], [52]. Reduced concentration of Zn in Cd-treated plants was also found by Balen et al. [9] and Cakmak [17], confirming Cd as an antimetabolite of Zn [53]. However, in our experiment, Zn content was still much higher in combined treatments than in control, or of the similar value; therefore, Zn deficiency can be excluded. Different responses in accumulation and interaction of investigated metals found in seedlings as well as in roots and leaves of adult plants could be a consequence of plant age and/or different metal distribution [54].

Treatments with Cd resulted in an increased content of MDA in all tissues compared to control, which was expected since it is well established that Cd in plant tissue induces oxidative stress that can damage membrane and consequently increase MDA content [6], [9], [46], [55]. Due to its chemical properties Cd cannot directly react with oxygen, but it can cause oxidative stress indirectly by inhibition of metabolic reactions [4]. In seedlings, obtained MDA content was mostly reduced after combined treatments with Zn in comparison to individual Cd treatment, suggesting alleviating effect of Zn. This result could be correlated with higher Zn uptake, which was observed in these combined treatments. Contrary, in roots and leaves of adult plants all combined treatments exhibited significantly higher MDA content in comparison to control. This result can be correlated with the findings that Zn had no significant effect on reduction of Cd content in leaves and even had a positive effect on Cd uptake in roots of plants exposed to combined treatments. Also, in both roots and leaves, higher Zn concentration (50 µM) significantly increased MDA content. Higher Zn concentrations can induce oxidative stress, which has already been reported for several plant species [9], [56], [57]. According to Bueno and Piqueras [58] ROS formation cannot be directly induced by Zn; namely, toxic forms of oxygen, which cause lipid peroxidation, are formed as a consequence of Zn interaction with lipid membrane which could produce conformational changes able to activate the plasma membrane-localized NADPH-oxidase, which could generate ROS.

Tobacco seedlings exposed to both applied Cd concentration exhibited increased carbonyl content, which can be considered as an evidence of the protein oxidative modification [59]. This result is in agreement with findings that Cd increased the content of carbonyl groups in growing plants [10], [59]. Among several metals, Cd has been shown to cause the depletion of protein bound thiol groups [60]. Application of Zn in combination with Cd reduced protein oxidation only when lower Cd concentration was applied, while Zn could not alleviate the effect of 15 µM Cd. In roots, exposure to both individual Cd treatments as well as combined treatments with Zn resulted with elevated carbonyl content. This could be correlated with high Cd accumulation, which was 25 times higher in roots than in leaves of tobacco plants. Also, higher Zn concentration significantly increased protein carbonyl content confirming that higher Zn causes oxidative stress and could be toxic for tobacco roots. Unlike roots, leaves responded with significantly enhanced protein oxidation only after exposure to combined treatments. These findings are in accordance with the results reported for *L. minor*, where significantly enhanced protein damage was also observed in plants treated with combination of Cd and Zn [9].

Alkaline protocol of the plant comet assay is a reliable and simple method which is widely used for estimation of the extent of induced DNA damage [61]. In our study, elevated DNA damage was recorded in all investigated tobacco tissues after the treatment with either individual Cd concentrations indicating its genotoxic effect, which was already reported for several plant species [9], [38], [62]. The possible pathway(s) of Cd induced genotoxicity are still unknown, but may involve the interaction of this metal with DNA, either directly or indirectly via the induction of oxidative stress [63]. Increased DNA damage in correlation with increased MDA and protein carbonyl contents, found in Cd-treated tobacco suggests that the overproduction of ROS induced DNA damage. Prominent DNA damage was also recorded in leaves after treatments with combinations of Cd and Zn, which can be correlated with elevated MDA, carbonyl and Cd content. On the other hand, in roots, no DNA damage was recorded after combined treatments, despite increased oxidative stress. This may indicate that Zn addition could protect DNA from Cd-induced damage as previously suggested [5], [64]. However, in seedlings exposed to combinations as well as individual Zn treatments increased DNA damage was found, although oxidative stress was not prominent, which suggests that high metal content (Cd and/or Zn) might also be involved in DNA damage due to their interaction with DNA [63]. Unfortunately, the comet assay, being a quantitative method, can not explain the mechanism underlying metal genotoxicity. For a better understanding of genotoxic effects of heavy metals other assays and endpoints (somatic mutations, chromosome aberrations, or micronuclei) should be performed [33], [38].

In response to the Cd-induced oxidative stress, plants employ antioxidative defense system to scavenge ROS and prevent destructive oxidative reactions [6], [8]. In our study, elevated PPX and APX activities were found in tobacco seedlings as well as in leaves and roots of adult plants exposed to Cd treatments. PPX and APX are known to play an important role in reducing oxidative stress by catalyzing the reduction of H_2_O_2_ [65]. Supplementation with Zn mostly decreased their activities, which in seedlings and roots can be correlated with reduced oxidative stress, thus, indicating that Zn could protect the phospholipids and proteins from thiol oxidation and disulfide formation by binding to the -SH groups of the membrane protein moiety [12]. Moreover, seedlings exposed to individual Cd treatments revealed the highest CAT activity, which was somewhat reduced after the addition of Zn. This is in a good correlation with PPX and APX results as well as with reduction of MDA and carbonyl content obtained in combined treatments, which once again confirms that Zn contributes to stability of the enzymes, membrane proteins and the lipid structure. In leaves, investigated metals had no influence on CAT activity. CAT also eliminates H_2_O_2_, but due to low substrate affinity it is less efficient than PPX in H_2_O_2_ scavenging [66]. However, in tobacco roots CAT activity was significantly reduced in all applied treatments, which indicates CAT suppression by binding of both metals on important groups of the enzyme [1]. It has already been reported that environmental stresses cause either enhancement or depletion of CAT activity, depending on the intensity, duration, and type of the stress [60].

In leaves, GR activity levels where unaffected by individual Cd treatments, while seedlings and roots revealed significantly lower values in comparison to control. Similar decrease in GR activity was also found in *Ceratophyllum demersum* [12] and tomato [15] in response to Cd stress. Zn supplementation failed to show impairment of GR activity. Moreover, individual Zn treatments in roots exhibited similar effects as Cd and resulted with significantly lower GR activity compared to control, which suggests that both metals had inhibitory effects on this enzyme.

A decrease in SOD activity was recorded in seedlings and roots exposed to individual treatments with both metals as well as their combinations. A reduction in SOD activity in plants exposed to metals has been attributed to an inactivation of the enzyme by H_2_O_2_ that is formed in different cellular compartments where SOD catalyses the scavenging of superoxide radicals [67]. However, leaves revealed a decreased SOD activity mostly after exposure to individual Cd treatments. A variable response of SOD activity was already reported for plants exposed to different metals, whose effects are showed to be dependent on the applied concentration, period of exposure and tissue type [67], [68], [69].

To evaluate the differences in induced biochemical responses in different tobacco tissues exposed to Cd and Zn and to identify the differences in responses after exposure to individual metals and metal mixtures, PCA was performed. PCA applied to the combined seedlings, roots and leaves data sets showed three clusters, suggesting varying responses of different tobacco tissues to metal-induced stress. However, there was no obvious subgrouping, indicating that difference in responses was primarily a result of tissue differences and not due to applied metal treatments. Roots responded with the most prominent oxidative stress and induction of PPX and APX which correlated with Cd and/or Zn accumulation. As mentioned before, in order to exclude metals from sensitive metabolism in the leaves, plants accumulate metals in roots, which could cause oxidative damage and induction of antioxidative enzymes [60]. Leaves were characterized with high MDA content, which corresponded to high SOD and CAT activity, implicating enhanced oxidative stress as well as antioxidative activity. High values of these parameters obtained in control leaves suggest that this could be related not only to metal induced stress but also to photosynthetic activity, which results in ROS production [70]. In seedlings, the most prominent response was DNA damage and inhibition of GR and SOD activity. However, when PCA analysis was done for each tissue separately, differences between metal treatments were noticed. In seedlings and roots, individual Cd treatments differed from others as well as control, exhibiting Cd-induced oxidative stress (changes in carbonyls, MDA content and APX activity), which correlated with lower Zn content. All combined treatments were grouped together with individual Zn treatments based on high Zn accumulation and less prominent oxidative stress, indicating that Zn could alleviate Cd-induced oxidative stress. However, this cluster also separated from control and was characterized with DNA damage (in seedlings) and lower GR and SOD activity (in both seedlings and roots), implicating that Zn accumulated in an amount which induced toxic response. On the other hand, different pattern was observed in leaves; due to high Cd accumulation and Cd-induced oxidative stress, treatments with Cd, applied either alone or in combinations with Zn, differed from treatments which did not contain Cd. It seems that in this tissue Zn did not accumulate in an amount that exerted toxic effects. Moreover, combined treatments separated from individual Cd treatments based on higher Zn uptake which correlated with somewhat higher SOD activity. Aravind and Prasad [5] also reported that Zn exposure enhanced SOD activity in *C. demersum*, which could be associated with possible role of Zn in stimulating the biosynthesis of antioxidant enzymes [17].

Obtained results suggest that metal treatments had greater impact on adult plants, particularly roots, compared to seedlings. The physiological responses of adult plants and young seedlings on abiotic stress may differ due to differences in the mechanisms that control the performance of the various stages of the life cycle [71]. Wang et al. [72] found that the tolerance to heavy metals may differ between young seedlings and adult plants, while Lefèvre et al. [31] reported that the physiological strategies of heavy metal tolerance were dependent on the age of the plants and the nature of the metal.

## Conclusions

In this study, Cd and Zn influenced the uptake of each other and revealed synergistic and/or antagonistic effects depending on the tissue type. The greatest impact of Cd- and Zn-induced stress was recorded in roots of adult plants. Analysing each tissue separately, results showed that in seedlings and roots individual Cd treatments generated oxidative stress, while in combined treatments it was less prominent, indicating that Zn could alleviate oxidative damage. However, DNA damage found in seedlings as well as lower GR and SOD activity recorded in both seedlings and roots, exposed to individual Zn treatments, implicate that higher Zn uptake also induced toxic effects. On the other hand, in leaves, treatments with Cd, applied either alone or in combination with Zn, induced oxidative stress, suggesting that in this tissue Zn did not have alleviating effects. Obtained results indicate that Cd-Zn interactions are tissue-dependent, which could result in activation of different mechanisms involved in the protection against metal stress.
